# Differentiation of Human Pluripotent Cells into Pancreatic Beta Cells for Disease Modeling and Cell Replacement Therapy for Diabetes

**DOI:** 10.3390/ijms26178749

**Published:** 2025-09-08

**Authors:** Anna A. Barinova, Alexandra Y. Bogomolova, Alexandra N. Bogomazova, Alyona A. Borisova, Sergey L. Kiselev, Alexandra V. Panova

**Affiliations:** 1 Lopukhin Federal Research and Clinical Center of Physical-Chemical Medicine, 119435 Moscow, Russiaabogomazova@rcpcm.org (A.N.B.); 2 Institute of Gene Biology, Russian Academy of Sciences, 119334 Moscow, Russia; 3 Vavilov Institute of General Genetics, Russian Academy of Sciences, 117971 Moscow, Russia; sl_kiselev@yahoo.com

**Keywords:** iPSC, diabetes, DM Type 1, insulin

## Abstract

Diabetes mellitus (DM) is a metabolic disease characterized by persistent hyperglycemia, resulting from defects in insulin secretion or impaired insulin action. In cases of severe pancreatic cell dysfunction and deficiency, the primary treatment remains lifelong insulin injections. A potential alternative is allogeneic pancreatic cell transplantation from a donor, which can stabilize glucose levels. However, the scarcity of donor material and the risk of immune rejection limit the widespread use of this approach. An alternative solution involves using in vitro-derived insulin-producing cells generated through the differentiation of pluripotent stem cells (PSCs), which could overcome the shortage of transplantable material. Furthermore, patient-specific cells—obtained directly from the patient via reprogramming of blood or skin cells into induced pluripotent stem cells (iPSCs)—would avoid immune rejection. Advances in this field have led to the active development and optimization of PSC differentiation into hormone-producing cells worldwide, with more than hundred patients dosed in clinical trials with ESC-derived cells and the single trial of iPSC-derived cells. This review highlights recent progress and prospects in generating insulin-producing cells from human PSCs, their applications in therapy development and disease modeling, as well as the current challenges and potential solutions.

## 1. Introduction

Diabetes mellitus (DM) is a metabolic disorder characterized by chronic hyperglycemia resulting from impaired insulin secretion, defective insulin action, or both [1]. Over the past three decades, the global prevalence of DM has surged fourfold, establishing it as the ninth leading cause of reduced life expectancy worldwide [2].

According to the International Diabetes Federation (IDF), approximately 425 million adults aged 20–79 years were living with DM in 2017, with an additional 325 million at high risk of developing the disease. Alarmingly, DM-related mortality had already surpassed 4 million annual deaths at that time. Current projections indicate a continued upward trajectory, with the number of affected individuals expected to rise to 629 million by 2045 [3].

Diabetes mellitus (DM) manifests through distinct pathogenic mechanisms. In type 1 diabetes (T1DM), autoimmune destruction of pancreatic β-cells leads to absolute insulin deficiency. In type 2 diabetes (T2DM), prolonged hyperglycemia causes both functional decline of insulin-producing β-cells and systemic insulin resistance [1].

Contemporary diabetes research focuses on leveraging the endocrine system’s natural physiology to develop therapies that closely mimic physiological insulin secretion. Current efforts prioritize enhancing the efficacy and safety of exogenous insulin therapy, which remains the cornerstone of DM treatment, through the advanced insulin formulations and novel delivery systems (e.g., aerosolized and oral insulin); improving pancreatic/islet cell transplantation techniques; developing in vitro-derived β-cell alternatives.

## 2. The Importance of Generating Insulin-Producing Cells

Given that diabetes fundamentally results from the destruction or dysfunction of pancreatic β-cells, effective treatment requires restoring normoglycemia by replenishing functional β-cell mass. Currently, several approaches exist for endocrine replacement therapy. Whole pancreas transplantation remains the gold standard for re-establishing physiological insulin secretion [4]. Alternatively, islet transplantation offers a minimally invasive alternative, implanting donor-derived β-cells capable of endogenous glucose regulation. This procedure has evolved over decades—from initial islet isolation attempts to successful Phase III clinical trials (2016)—and has been performed in over 1500 patients worldwide since 2000 [5]. Notably, islet transplantation is now recognized as one of the safest procedures in transplantology, combining therapeutic efficacy with reduced invasiveness.

The Edmonton protocol remains one of the most successful approaches for islet cell isolation and transplantation. According to 2016 multicenter trial results, this procedure achieved normoglycemia in 87.5% of patients at 1 year and 71% at 2 years post-transplantation [5]—a significant improvement over 2006 outcomes where just 31% maintained insulin independence after two years [6].

The protocol involves infusion of the islet cell suspension into the portal vein (which delivers the cells directly to the liver), followed by an immunosuppressive regimen combining etanercept, simulect, and rapamycin [7].

Clinical studies have documented various complications associated with donor islet transplantation. The most significant and potentially limiting complications include (1) sensitization to donor HLA antigens, which requires lifelong immunosuppressive therapy, and (2) increased risk of malignant neoplasms due to prolonged immunosuppression. Reported adverse outcomes—including transplant rejection, development of basal or squamous cell skin carcinomas, and fatal cases—occur in ≤3% of recipients [7,8]. These risks, combined with the limited availability of cadaveric islet cells, have driven the search for alternative sources of functional pancreatic cells for transplantation.

Human pluripotent stem cells (PSCs) represent one promising source of specialized cells that can be generated ex vivo in laboratory conditions. Currently, researchers are developing various micro- and macro-encapsulated implants derived from pancreatic endoderm obtained through directed differentiation of human embryonic stem cells (ESCs). These implants, designed for patients with autoimmune type 1 diabetes (T1DM), include some that have already entered clinical trials. A notable example is the pioneering PEC-Encap™ (also known as VC-01™) by ViaCyte [9].

Preclinical studies in mice demonstrate that such encapsulated implants can mature into functional grafts post-transplantation, serving as a source of biologically active β-cells for T1DM patients for at least one year [10]. Over the past decade, differentiation protocols for converting human PSCs into pancreatic cells and their transplantation methods have achieved remarkable progress. Contemporary PSC differentiation techniques into insulin-producing cells rely on our understanding of mammalian pancreatic and islet cell embryogenesis, resulting in sophisticated, multi-stage protocols.

## 3. Structural Organization of the Pancreas and Its Embryogenesis

**Figure 1:**
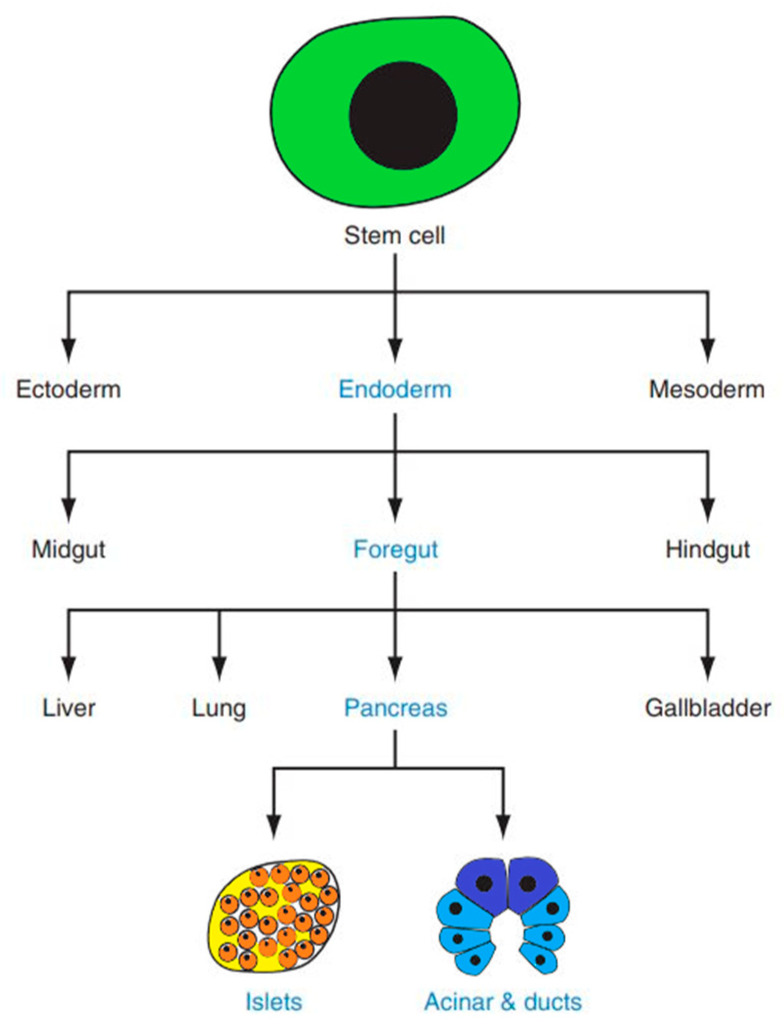
Pancreatic lineage specification from embryonic stem cell progenitors [12].

Insulin is produced by pancreatic beta cells. The pancreas itself is a complex organ originating from two endodermal primordia—the dorsal and ventral buds—which emerge on opposite sides of the distal foregut (Figure 1) [11]. The mature pancreas comprises two functionally and structurally distinct components: the exocrine and endocrine tissues. The exocrine compartment produces digestive enzymes, while the endocrine portion secretes metabolically active hormones [12].

The exocrine compartment, accounting for ~98% of the adult pancreas by mass, comprises acinar and ductal epithelial cells. In contrast, the endocrine portion is organized into specialized microstructures called the islets of Langerhans [12]. These islets harbor distinct cell populations that secrete key hormones: glucagon (α cells), insulin (β cells), somatostatin (δ cells), ghrelin (ε cells), and pancreatic polypeptide (PP cells).

Pancreatic β cells play a central role in glucose regulation by secreting insulin, which is essential for maintaining normoglycemia. These cells dynamically adjust insulin synthesis and release in response to fluctuations in blood glucose levels, thereby serving as a critical component of systemic glucose homeostasis [13].

The embryonic development of the pancreas progresses through three main stages. The first is the undifferentiated stage, during which endodermal protrusion initiates pancreatic morphogenesis. In humans, this occurs during the fourth week of gestation [14]. At this stage, expression of the transcription factor (TF) SSH decreases while PDX1 (pancreatic and duodenal homeobox 1) begins to be expressed. PDX1, also known as insulin promoter factor 1, represents the most significant and earliest marker of pancreatic progenitor cells. However, it is not a specific marker, as PDX1 expression contributes to the development of not only the pancreas but also the posterior stomach, duodenum, and bile ducts [11,15]. Concurrently, expression of PTF1a (pancreas-specific transcription factor 1a) is observed. Unlike PDX1, PTF1a expression in the endoderm is specific to pancreatic progenitor cells and does not initiate development of other digestive system organs [16].

The second stage involves formation of primary ducts, with progenitor islets beginning to separate, differentiate, and detach from the basement membrane. During this phase, both ventral and dorsal pancreatic primordia express the TFs SOX9, PDX1, and GATA-binding protein 4 (GATA4) [17,18].

In the final stage of human embryonic development, the pancreas undergoes substantial mass expansion through progenitor cell proliferation. Cellular heterogeneity becomes evident as centrally located SOX9+/NKX6.1+ cells within the islets show reduced GATA4 expression, while peripheral clusters of SOX9+/GATA4+/NKX6.1+ proacinar “tip” cells lose NKX6.1 expression within a week [11].

During mid-fetal development, NEUROG3 expression increases sharply, coinciding with the appearance of fetal β-cells—the first and predominant islet cell type to emerge [17]. SOX9 expression disappears in cells maintaining NEUROG3 levels and becomes undetectable in endocrine cells, though it persists in pancreatic ductal cells [11]

These developmental processes indicate that formation of the human β-cell pool is completed in utero at least 5 weeks (and likely 3 months) before birth. Consequently, subsequent expansion of fetal β-cell mass occurs primarily through proliferation of existing β-cells rather than differentiation from progenitor cells. Final maturation and enrichment of β-cells requires expression of the transcription factors MAFA and NKX6.1 [19].

## 4. Approaches for Generating Beta Cells from Human Pluripotent Stem Cells

Embryonic development and tissue formation originate from a cluster of cells within the day 5 blastocyst known as the inner cell mass (ICM). For in vitro studies, pluripotent cells can be derived through two primary approaches: isolation from the ICM of preimplantation embryos to establish embryonic stem cells (ESCs) and the reprogramming of adult somatic cells to generate induced pluripotent stem cells (iPSCs)

The establishment of mouse ESC lines dates back to the early 1980s [20]. Notably, human ESCs differ substantially from their murine counterparts, as they are typically derived from later blastocyst stages that correspond developmentally to the mouse epiblast stage, necessitating distinct culture conditions. The groundbreaking generation of iPSCs was first achieved through retroviral-mediated transduction of mouse embryonic and adult fibroblasts with four key transcription factors (Oct4, Sox2, Klf4, and c-Myc) that are naturally expressed in ESCs [21]. Subsequent research demonstrated that human iPSCs could be derived from various somatic cell types. Both ESCs and iPSCs possess the remarkable potential to differentiate into any cell type in the body, making them invaluable for fundamental biological research, development of cell-based therapies and disease modeling applications.

Early studies relied on spontaneous ESC differentiation through embryoid body formation, which generated cells representing all three germ layers—including endoderm—capable of both directed and spontaneous differentiation into various cell types, including insulin-producing cells. For directed differentiation, researchers supplemented culture media with additives like N2, B27, and nicotinamide. These components activated genes specifically expressed in pancreatic progenitor cells. Notably, their inclusion during embryoid body differentiation significantly enhanced cellular insulin secretion. Importantly, even undirected differentiation protocols without supplemental additives yielded a small population of pancreatic progenitor cells expressing lineage-specific markers [22]. However, these early studies and protocols were not sufficiently efficient in producing monohormonal mature insulin-secreting cells.

Advances in understanding murine pancreatic development have enabled novel approaches for differentiating human pluripotent stem cells (PSCs). Researchers have successfully recapitulated all key stages of pancreatic organogenesis in vitro using human ESC lines, including definitive endoderm formation, primitive gut tube development, pancreatic endoderm specification, endocrine progenitor cell differentiation.

Through sequential culture media supplementation with stage-specific factors, the differentiated cells achieved insulin secretion levels comparable to mature pancreatic islets. However, most resulting cells exhibited polyhormonal characteristics, co-expressing insulin, glucagon, somatostatin, C-peptide, and ghrelin [23]. Subsequent refinements in 2009 yielded pancreatic cells with enhanced functionality; 25% demonstrated glucose-responsive insulin and C-peptide secretion, mimicking adult human islet behavior. The majority expressed key β-cell markers (PDX1, NKX6.1, and MAFA) and these cells showed physiological glucose-stimulated insulin release [24].

**Figure 2:**
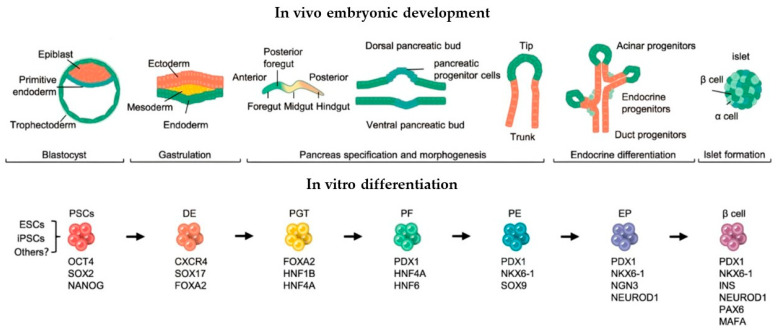
Stepwise differentiation of pancreatic β cells from human pluripotent stem cells. By recapitulating in vivo pancreatic development, human pluripotent stem cells (PSCs) can be directed through a stepwise differentiation process to generate pancreatic lineage cells, including functional β cells. Key stages: DE: Definitive Endoderm, PGT: Primitive Gut Tube, PF: Posterior Foregut, PE: Pancreatic Endoderm, EP: Endocrine Precursor (from [25] with modifications).

Multiple successful differentiation protocols have now been established for deriving insulin-producing cells from human dermal fibroblasts. These approaches share a common foundation in recapitulating key stages of pancreatic embryogenesis (Figure 2): sequential induction and the stage-specific addition of growth factors/small molecules [25].

The derivation of pancreatic progenitor cells and their subsequent in vivo maturation has demonstrated the ability to maintain glucose homeostasis in diabetic mice, revealing promising avenues for novel diabetes therapies. Notably, Kroon and colleagues demonstrated that in vitro-derived pancreatic endoderm transplants can secrete insulin and C-peptide at levels comparable to cadaveric islet transplantation. Furthermore, the engrafted insulin-producing cells exhibited multiple functional characteristics of mature β-cells [26]. Rezania and colleagues further demonstrated the potential of differentiated ESCs as an alternative to cadaveric islets for diabetes treatment. Following transplantation of pancreatic progenitor cells into immunodeficient diabetic mice, blood insulin levels progressively increased and human C-peptide secretion became regulated by food intake and glucose levels. During in vivo maturation, the ESC-derived endocrine cells exhibited gene/protein expression profiles characteristic of developing human fetal pancreas [27]. Subsequent studies reported successful in vitro differentiation of human ESCs into pancreatic progenitors. Post-transplantation in mice up to 90% of cells differentiated into islet-like clusters, and 40–50% acquired glucose-responsive insulin secretion capacity. These functional cells expressed definitive β-cell markers (PDX1 and NKX6.1) [28].

**Figure 3:**
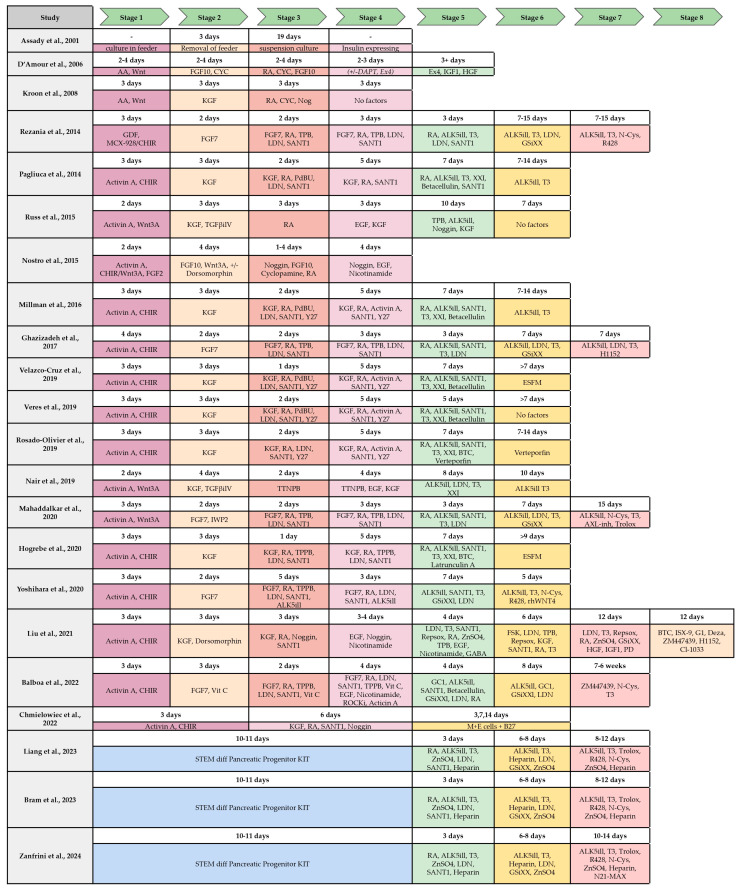
Comparison of published protocols of differentiation of hPSC to insulin-producing beta-cell by stages and molecules/growth factor used. S1–S7, stages from 1–7 (from [31] with modifications).

Until 2014 attempts at generating insulin-producing (INS+) cells from human pancreatic progenitors in vitro have generated cells with immature or abnormal phenotypes. These cells either fail to perform glucose-stimulated insulin secretion in vitro, fail to express appropriate beta-cell markers such as NKX6.1 or PDX1, abnormally coexpress other hormones like glucagon (GCG), fail to function after transplantation in vivo, or display a combination of these abnormal features. Figure 3 summarizes the majority of the protocols developed for the beta-cell differentiation, the stages and the factors and molecules used for differentiation.

In 2014, Pagliuca and colleagues reported a breakthrough protocol for large-scale production of functional human β-cells from iPSCs in vitro. Through sequential modulation of multiple signaling pathways in 3D culture (embryoid body-like structures), without using transgenes or genetic modifications, they successfully generated glucose-responsive, monohormonal (insulin-producing) fully differentiated β-like cells. The derived cells exhibited all hallmark features of native β-cells, including co-expression of key β-cell markers, characteristic β-cell ultrastructure and functional equivalence to primary islet β-cells in both in vitro and in vivo assessments [29]. These findings demonstrate the strong therapeutic potential of these iPSC-derived β-cells for diabetes treatment.

In 2014, Rezania and colleagues developed an enhanced seven-step in vitro differentiation protocol building upon existing methods for pancreatic progenitor generation. Their key findings at Early Differentiation revealed that Vitamin C supplementation during initial stages yielded Pdx1+/NKX6.1+ pancreatic progenitors and these progenitors showed low expression of Ngn3 and related genes. During the progenitor maturation a specific combination of supplements (ALK5 inhibitor, BMP receptor inhibitor, and T3) promoted increased Ngn3 expression and generation of cell populations expressing PDX1, NKX6.1, NEUROD1, and NKX2.2. During the endocrine commitment the continued treatment with ALK5 inhibitor, BMP receptor inhibitor, T3, and γ-secretase inhibitor produced monohormonal PDX1+/NKX6.1+/NEUROD1+ cells with the Insulin+/glucagon-/somatostatin- phenotype. Final Maturation screening identified R428 (AXL inhibitor) as critical for MAFA induction in committed β-like cells and functional maturation. The resulting cells exhibited hallmark features of mature β-cells: glucose-stimulated insulin secretion and the rapid glycemic correction in diabetic mouse models [19].

For example, in 2016, Zhu and colleagues successfully differentiated fibroblasts into pancreatic progenitors. The study employed non-integrating episomal reprogramming factors (OCT4, SOX2, KLF4, and short hairpin RNA) combined with growth factors (including epidermal growth factor (EGF) and fibroblast growth factor (FGF)) and various chemical compounds. This approach was termed CASD—the Cell-Activation and Signaling-Directed lineage conversion strategy. During in vitro reprogramming and differentiation, researchers obtained β-cell progenitors and functional pancreatic β-like cells expressing all key transcription factors characteristic of this cell type. When these β-like cells were transplanted in vivo into mice with induced diabetes, they demonstrated glucose-dependent insulin secretion capable of fully compensating for the animals’ hormonal deficiency. Using this method, the laboratory generated not only functional β-cells but also cardiomyocytes, neural progenitors, angioblast-like progenitors, endothelial cells, and hepatocytes [30].

In 2016, Millman and colleagues compared β-cells derived from skin fibroblasts of patients with T1DM and healthy individuals both in vitro and in vivo. The resulting cell lines were nearly indistinguishable in their glucose-stimulated insulin production, expression of key β-cell markers and response to antidiabetic drugs. The study revealed that when β-cells from T1DM patients were subjected to chemical stress, they lost β-cell marker expression. These cells also showed susceptibility to cytokine stress, though Alk5i treatment provided partial protection. However, complete assessment of the cell lines’ identity would require several years of in vivo observation, as diabetes development and β-cell destruction are prolonged processes [32].

The described protocols involve differentiation of adherent PSC cultures followed by aggregate formation during beta cell maturation stages. However, alternative approaches proposing differentiation through embryoid body formation at the initial stage have been developed—the authors established culture conditions that closely mimic islet organogenesis and beta cell maturation in the pancreas. The strategy focused on generating endocrine cell clusters in vitro through isolation and reorganization of immature beta-like cells, ultimately producing enriched beta clusters comparable in size to individual islets of Langerhans. Research demonstrated that these beta clusters exhibit physiological properties similar to native human islets, like stable insulin secretion, calcium channel signaling in response to secretagogues and enhanced mitochondrial energy production. Notably, endocrine cell clustering promotes metabolic maturation by activating cellular respiration processes essential for stimulated insulin secretion in mature beta cells. When transplanted into mice, the beta clusters demonstrated glucose-responsive insulin secretion within just three days. These findings show that replicating endocrine cell clustering enables derivation of stem cell-derived beta cells that closely resemble their endogenous counterparts [33].

To enhance the differentiation efficiency of iPSCs into pancreatic β-cells, researchers identified specific chemical compounds that both improve differentiation yield and stabilize pancreatic progenitor aggregates. The optimized protocol incorporated forskolin during endocrine precursor induction, ISX-9, G-1, 3-Deazaneplanocin A, ZM447439, and CI-1033 at the final maturation stage. The resulting β-like cells demonstrated both safety and functional efficacy in vivo, successfully engrafting and maintaining glycemic control when transplanted into immunodeficient diabetic mice [34].

In a 2022 study, researchers conducted functional characterization of iPSC-derived β-cell aggregates. The results demonstrated that aggregates generated using microwell technology exhibited greater glucose sensitivity in vitro compared to those formed under dynamic suspension culture conditions. However, both aggregate types showed reduced glucose-stimulated insulin secretion relative to human β-cell lines, indicating their immature phenotype. For in vivo assessment, transplantation under the renal capsule of immunodeficient humanized mice revealed the progressive β-cell maturation over 14 weeks, development of normal glucose responsiveness and maintenance of normoglycemia after ablation of endogenous β-cells (unlike graft-free controls that developed diabetes) [35]. In a related 2023 study, Liang demonstrated that static (versus dynamic) aggregate formation enhanced both the efficiency and uniformity of islet maturation in vitro [36].

Thus, following the groundbreaking approaches developed by two laboratories in 2014, the paradigm for differentiating PSCs into pancreatic β-cells shifted dramatically, enabling the generation of cells capable of glucose-dependent insulin secretion. To date, ongoing protocol optimizations continue to refine the process, progressively aligning in vitro differentiation with embryonic developmental pathways.

## 5. Clinical Applications and Disease Modeling Using PSC-Derived β-Cells

The ability to generate functional beta cells from human pluripotent stem cells (PSCs) has opened unprecedented opportunities for disease modeling. While the T1DM pathogenesis is well-studied, the inherited forms of diabetes, such as neonatal diabetes and maturity-onset diabetes of the young (MODY), remain unexplored. Many of these monogenic disorders were previously poorly understood due to limited access to patient-specific pancreatic tissue and the challenges of recapitulating human disease mechanisms in animal models. PSC-derived beta cells now allow us to recreate disease phenotypes in vitro by introducing patient-specific mutations using genome editing tools like CRISPR-Cas9.

Some monogenic forms of diabetes can result from mutations in the INS gene. Ma and colleagues conducted a study to correct the homozygous INS ATG> ATA mutation at the translation start site using CRISPR/Cas9. As part of the experiment, both the mutant line and the corrected codon line were differentiated into pancreatic endocrine cells. Despite insulin being a defining protein of β-cells, differentiation of the mutant cells yielded an INS- line that still expressed key markers such as PDX1, MAFA, and NKX6.1. These findings suggest that insulin is not required for the formation of pancreatic endocrine cells. Furthermore, such insulin-negative β-cell precursors could be valuable for future research, including studies on the role of insulin in endoplasmic reticulum (ER) stress-induced β-cell destruction and the investigation of insulin epitopes in autoimmune diabetes [37].

Overall, editing mutations in patient-derived iPSC lines enables the creation of isogenic models for studying monogenic forms of diabetes. These models are being increasingly utilized today and help elucidate the pathogenesis of monogenic diabetes with heterozygous mutations. According to current knowledge, such mutations should not cause severe disease, yet they result either in maturity-onset diabetes of the young (MODY) or in earlier and more severe forms—neonatal diabetes. For instance, iPSCs carrying insulin gene mutations that cause severe neonatal diabetes provide valuable insights into disease development mechanisms [38,39].

To investigate how proinsulin molecular conformation affects β-cell proliferation and apoptosis, Balboa and colleagues employed iPSC-derived model β-cells. They reprogrammed skin fibroblasts from a patient carrying clinically significant INS gene missense mutations (C96R and C109Y), generated isogenic controls using CRISPR-Cas9 to correct these mutations and differentiated both corrected (INS+) and mutant (INS-) lines into β-cell precursors for further comprehensive in vitro and in vivo analyses. The mutant cell line had impaired insulin secretion and enhanced ER stress. Altered mTORC1 (mechanistic target of rapamycin complex 1) signaling and reduced respiratory chain subunit expression was identified. Notably absent apoptosis was detected despite these abnormalities. The authors concluded that misfolded proinsulin induces ER stress, disrupts mTORC1 pathway signaling, impairs cellular respiration and inhibits proliferation without triggering apoptosis. This proliferation defect ultimately reduces normal INS+ β-cell populations, contributing to neonatal diabetes development. Theoretically, these findings could inform novel neonatal diabetes treatment strategies targeting transient mTORC1 stimulation during early disease stages [38].

The combination of genome editing technology and the ability to derive beta cells from patient-specific iPSCs with monogenic forms of diabetes opens future possibilities for using cell replacement therapy to treat these disorders. In the aforementioned study, iPSCs carrying edited mutations were differentiated into beta cells and transplanted into the leg muscle of immunodeficient mice with destroyed pancreatic islets. Following transplantation, glucose-dependent C-peptide was detected at normal levels in the mice’s blood, resulting in normalized blood glucose concentrations [37].

Panova and colleagues used iPSCs from a patient diagnosed with neonatal diabetes mellitus carrying the INS mutation in the second intron (c.188-31G>A) and engineered isogenic CRISPR/Cas9 mutation-corrected cell lines. Differentiation into beta-like cells demonstrated that mutation led to the emergence of an ectopic splice site within the INS and appearance of the abnormal RNA transcript. Isogenic iPSC lines differentiated into beta-like cells showed a clear difference in formation of organoids at the pancreatic progenitor stage of differentiation. Cells expressing mutated insulin demonstrated significant decrease in proliferation capacity and activation of ER stress and unfolded protein response (UPR)-associated genes. These findings shed light on the mechanism underlying the pathogenesis of monogenic diabetes [39].

Whether using cadaveric material or differentiated hormone-producing cells for pancreatic transplantation, an easily accessible and surgically safe site will inevitably be selected for implantation. While such localization of the new hormone-secreting tissue may prove functionally effective, it remains physiologically unnatural due to the absence of proper cellular microenvironment, lack of secretory regulation by neighboring cells and the insufficient vascularization. As an alternative approach, researchers have proposed generating beta cells from their developmentally closest relatives—glucagon-producing alpha cells that naturally reside within the same islets of Langerhans [40]. The study employed an adeno-associated virus (AAV) carrying Pdx1 and MafA genes to reprogram alpha cells into functional beta cells. Transgene delivery resulted in normalization of glucose levels in toxin-induced diabetic mice and glycemic correction in autoimmune NOD mice. Euglycemia and the newly formed insulin-producing cells persisted in NOD mice for 4 months before autoimmune diabetes recurrence. This approach also demonstrated efficacy in human islet cells, restoring normoglycemia in NOD/SCID mice following transplantation.

To date, few clinical trials have been conducted using pluripotent stem cells (PSCs) differentiated into insulin-producing cells, with nearly all involving products developed by ViaCyte and Vertex Pharmaceuticals. Table 1 summarizes all clinical trials involving transplantation of human PSC-derived beta cells. These trials share several key characteristics; they all focus on type 1 diabetes mellitus (T1DM) treatment and utilize allogeneic cells, specifically human embryonic stem cells (ESCs), as the differentiation source. They have varied immunosuppression protocols, as some employ encapsulated cell products requiring no immune suppression.

ViaCyte pioneered the development of human pluripotent stem cell (PSC)-derived β-cells for transplantation with their PEC-01 product. Preclinical studies demonstrated that encapsulated β-cell grafts maintained functionality for ≥2 years in murine models, achieving successful vascularization and restoration of normal metabolic parameters [10]. An open-label Phase 1/2 clinical trial STEP ONE evaluated the safety, tolerability, and efficacy of the PEC-Encap product candidate. Cohort 1 of the study, which was designed to deliver a sub-therapeutic dose of cells, has enrolled 19 patients with established but stable type 1 diabetes. Patients were implanted subcutaneously with two different PEC-Encap unit sizes: VC-01-250 and VC-01-20. The larger VC-01-250 units are used primarily to evaluate safety and tolerability, and eventually efficacy, while VC-01-20 units are smaller “sentinels” that are removed at various time points throughout the study for histological and other analyses. PEC-Encap units were explanted at approximately 1, 2, 4, 8, 12, 16, and 104 weeks (two years). After all units were removed, patients continued in a follow-up observational study. Interim data (12 weeks to 2 years) demonstrated that PEC-Encap was well-tolerated, with AEs primarily linked to surgery/post-op care, not off-target growth detected. The Encaptra system prevents allo-/autoimmune rejection, with no donor-specific antibodies, autoantibody increases, or T-cell sensitization. Despite suboptimal engraftment due to foreign body reactions, differentiated insulin+ β-cells and glucagon+ α-cells persisted for up to 2 years in well-vascularized regions, without immunosuppression [51].

In 2021 Shapiro et al. report preliminary proof-of-concept that in 17 people with type 1 diabetes, pancreatic endoderm cells in an investigational subcutaneous device (VC-02) achieved engraftment and insulin expression in 63% of units at 3–12 months post-implant. Pluripotent stem cells may be a scalable, renewable alternative to pancreatic islet transplants [45].

Phase 1/2 multicenter trial (NCT03163511) investigated whether enhanced cell dosing (2–3× higher) and optimized device membrane architecture could improve engraftment. Interim 12-month data from one cohort (*n* = 10) revealed that three of ten initially C-peptide-negative patients sustained levels ≥ 0.1 nmol/L from month 6 onward. These responders showed significant improvement in CGM-measured time-in-range (up to 85% from 55% baseline in the top responder) and reduced exogenous insulin requirements. Histological analysis of explanted devices in the highest responder (peak C-peptide 0.23 nmol/L) showed 4% β-cell survival at 6 months, suggesting avenues for protocol optimization [47].

In 2024 Vertex announced positive results from ongoing phase 1/2 study of VX-880 for the treatment of type 1 diabetes. VX-880 is an investigational stem cell-derived, fully differentiated islet cell therapy, in people with type 1 diabetes (T1D) with impaired hypoglycemic awareness and severe hypoglycemic events (SHEs). At baseline, all patients in the study had undetectable fasting C-peptide (a marker of endogenous insulin secretion), a history of recurrent SHEs in the year prior to screening, and required an average of 39.3 (min, max; 19.8, 52.0) units of insulin per day. Following a single infusion of VX-880 at the full dose, all 12 patients demonstrated islet cell engraftment and glucose-responsive insulin production by day 90. Eleven of twelve patients reduced or eliminated use of exogenous insulin. Three patients with at least 12 months of follow-up met the primary endpoint of elimination of severe hypoglycemic events (SHEs) with HbA1c < 7.0%, and the secondary endpoint of insulin independence [49]. In 2025 VX-880 initiated the Phase 3 portion of the Phase 1/2/3 study. This pivotal trial is well underway and on track to complete enrollment and dosing in the first half of 2025, setting up global regulatory submissions in 2026. VX-880 has previously been granted Fast Track designations from the U.S. Food and Drug Administration.

Also, in 2025 Vertex completed enrollment and dosing of the Phase 1/2 study for its encapsulated cell therapy VX-264 (cells + device), including the planned Day 90. Participants received the full dose of the investigational therapy, consisting of fully differentiated pancreatic islet cells enclosed in Vertex’s proprietary immunoprotective device. The study evaluated two primary endpoints—safety and change in peak C-peptide levels during a mixed-meal tolerance test (MMTT) at Day 90 compared to baseline. While VX-264 demonstrated an acceptable safety profile, it did not meet the efficacy endpoint, as C-peptide levels did not increase sufficiently to provide clinical benefit. As a result, Vertex will not advance VX-264 into further clinical trials. The company plans additional analyses, including examination of explanted devices, to further investigate these outcomes [52].

While all the previously mentioned therapies were developed using human embryonic stem cells (ESCs), so far, only a single patient has been treated with transplanted beta cells generated from their own induced pluripotent stem cells (iPSCs) [50].

A pioneering first-in-human Phase I clinical trial (ChiCTR2300072200) has reported one-year follow-up data from a patient receiving autologous chemically induced pluripotent stem cell-derived islets (CiPSC-islets) for type 1 diabetes treatment. The patient achieved sustained insulin independence within 75 days post-transplantation, with dramatic metabolic improvements; time-in-range increased from 43.18% at baseline to 96.21% by month 4, while glycated hemoglobin stabilized at non-diabetic levels. Throughout the study period, the patient maintained exceptional glycemic control (>98% time-in-range and ~5% HbA1c). At the one-year mark, all primary endpoints were successfully met with no transplant-related complications observed. These promising initial results demonstrate the potential of CiPSC-islet transplantation and support further clinical investigation of this novel therapeutic approach for type 1 diabetes [50].

A critical limitation of beta cell transplantation—whether using donor islets or stem cell-derived beta cells—is the requirement for long-term immunosuppression to prevent immune rejection, particularly in autoimmune type 1 diabetes (T1DM). Despite the success of the 2024 case of autologous iPSC-derived islets restoring insulin independence [50], it is worth mentioning the fact that the patient required immunosuppressive therapy to protect the graft from autoimmune destruction. This is a recurring issue in clinical trials, including Vertex’s VX-880 and earlier islet transplantation protocols like the Edmonton regimen, where immunosuppression can introduce risks of infections, malignancies, and metabolic complications. Although encapsulation strategies aim to circumvent immune rejection, their efficacy remains inconsistent, as seen with Vertex’s discontinued VX-264 trial. For stem cell-derived therapies to become widely viable, overcoming the need for immunosuppression—through immune-evasive engineering or improved encapsulation—must be a central focus.

## 6. Conclusions

This review explores current strategies for differentiating human pluripotent stem cells (PSCs) into functional pancreatic β-cells—a promising therapeutic approach for diabetes mellitus (DM). Recent breakthroughs include the development of multi-stage differentiation protocols that recapitulate critical phases of embryonic pancreatic development. The successful generation of glucose-responsive, insulin-secreting cells marks substantial progress toward alternatives to conventional treatments like insulin injections or donor islet transplantation.

A major advancement has been the establishment of scalable methods for producing functional β-cells from human PSCs, enabling personalized therapy. Clinical trials by ViaCyte and Vertex Pharmaceuticals have demonstrated the safety and efficacy of encapsulated β-cell implants, though further optimization is needed to enhance cell survival and long-term function. Nevertheless, several challenges persist, including immune rejection risks, functional immaturity of in vitro-derived β-cells, potential tumorigenicity and need for improved encapsulation and delivery systems to maintain long-term viability.

Future research priorities include developing immune-evasive approaches, such as gene-edited “immune-stealth” cells that circumvent the need for immunosuppression. PSC-derived β-cells hold tremendous potential for revolutionizing diabetes treatment, potentially offering durable or curative solutions for T1DM, T2DM, and monogenic diabetes—particularly where traditional therapies prove inadequate. Key focus areas for advancement include refinement of differentiation protocols, optimization of transplantation techniques and the risk mitigation strategies for stem cell applications.

## Figures and Tables

**Table 1:** Clinical trials using the pancreatic cells derived from human PSCs.

Scheme	Drug	Study Start	Trial Number	No of Patients Dosed	Ref.
ViaCyte	VC-01 (pancreatic endoderm cells (PEC-01) in Encaptra device)	2014	NCT02239354	19	[41,42,43]
		2019	NCT04678557	31	
	VC-02 (pancreatic endoderm cells (PEC-01) in macroencapsulation device)	2017	NCT03162926	3	[42,43,44,45,46,47]
		2017	NCT03163511	49	
	VCTX210A (gene-edited pancreatic endoderm cells in macroencapsulation device)	2022	NCT05210530	7	[48]
Vertex Pharmaceuticals	VX-880 (pancreatic islet cells)	2021	NCT04786262	16	[49]
	VX-264 (pancreatic islet cells VX-880, encapsulated)	2023	NCT05791201	17	–
Tianjin First Center Hospital	CiPSC islets (islet-like cells from iPSC)	2023	ChiCTR2300072200	1	[50]
